# Developing research priorities for palliative care in Colombia: a priority setting partnership approach

**DOI:** 10.1186/s12904-024-01534-z

**Published:** 2024-08-01

**Authors:** Tracey McConnell, Cindy V. Mendieta, Esther de Vries, Jose A. Calvache, Gillian Prue, Sam Ahmedzai, Joanne Reid

**Affiliations:** 1 Marie Curie Hospice Belfast, Belfast, UK; 2 School of Nursing and Midwifery Queen’s University Belfast, Belfast, UK; 3 Department of Clinical Epidemiology and Biostatistics, Faculty of Medicine, Pontificia Universidad Javeriana, Bogota, Colombia; 4 Department of Nutrition and Biochemistry, Faculty of Science, Pontificia Universidad Javeriana, Bogota, Colombia; 5 Department of Anesthesiology, Faculty of Health Sciences, Universidad del Cauca, Popayan, Colombia; 6 Department of Anesthesiology, Erasmus University Medical Center Rotterdam, Rotterdam, The Netherlands; 7 Department of Oncology, The University of Sheffield, Sheffield, UK

**Keywords:** Palliative care, Research priorities, Nominal group technique, Priority setting partnership, Consensus methods

## Abstract

**Background:**

A recent Lancet commission called for more research on palliative care in low- and middle-income (LMIC) countries such as Colombia. A research priority setting approach has been recommended by The Global Forum for Health Research to address the huge gap in research output between LMIC and high-income countries, with influential health service bodies recommending the active involvement of non-research expert stakeholders in establishing research priorities to address service user needs.

**Method:**

Priority setting partnership (PSP) following the four stages of the James Lind Alliance methodology; establishing the partnership, identifying evidence uncertainties, refining questions and uncertainties, and prioritization. Data from MS forms were analysed using descriptive statistics.

**Results:**

A total of 33 stakeholders attended an online PSP workshop and completed the Mentimeter exercise in Microsoft Teams. A total of 48 attended the subsequent in person prioritisation exercise in urban Bogota (*n* = 22) and rural Popayan (*n* = 25). The stakeholders were a diverse group of health professionals (physicians, medical students, nurses, dentists, physiotherapists, nutritionist, occupational and speech therapists), financial and administrative staff and patients with life-limiting illness and caregivers. Top research priorities included patient and caregiver needs, service provider education and training, and better integration of palliative care with cancer and non-cancer services. The key challenges included a lack of interest in palliative care research, along with funding, time and resource constraints. Key solutions included collaboration across disciplines and settings, highlighting benefits of palliative research to help secure adequate resources, and multicentre, mixed method research, with patient involvement from the research development stage.

**Conclusion:**

The findings of this PSP should be disseminated among palliative care associations worldwide to inform international multicentre studies, and among governmental and nongovernmental organisations that promote research in Colombia. A focus on patient and family caregiver palliative care needs in Colombia should be prioritised.

**Supplementary Information:**

The online version contains supplementary material available at 10.1186/s12904-024-01534-z.

## Background

The World Health Organization (WHO) acknowledges access to Palliative Care (PC) as a human right for children, adults, and their families to prevent and relieve suffering from a range of life-limiting illnesses, from point of diagnosis to end-of-life [1]. PC is a holistic approach which assesses and treats the whole person’s physical, psychosocial, and spiritual needs [1]. Early access to PC for those with life limiting illness (cancer and non-cancer) improves symptoms and quality of life for patients and informal carers and reduces financial burden on both families and health systems [2–5].

However, inadequate access to PC persists worldwide, with only 14% of the global population receiving PC when needed [1]. A large proportion (estimated at 78% of adults) requiring PC reside in low- and middle-income countries (LMIC) such as Colombia, situated in South America [1]. Colombia has approximately 50 million residents, with 77% living in cities, 7.1% in small municipal settlements (villages) and 16% across scattered rural areas [6]. The Lancet Commission on Palliative Care and Pain Relief estimated up to 250,000 adults in Colombia require PC per year [7], yet a recent health policy and systems analysis show that PC services fall short of meeting these population needs [8], and most PC services are individual efforts, institution-based and centralized in the main cities. Although up to 83% of adults who died from chronic illnesses in Colombia required PC, up to 30% died without receiving it [9]. Some departments in Colombia, particularly those in the Orinoquia and Amazonia regions, lack well-developed palliative care (PC) services [9]. In contrast, most PC services are concentrated in three main areas: Bogotá D.C., the Center, and the Caribbean [10]. For paediatric patients, there are only ten hospital services and five outpatient services, primarily located in the main capital cities of Bogotá, Cali, Medellín, and Neiva [9]. Colombia is currently developing an action plan: ‘Building a positive environment: the road to palliative care—Colombia 2026’, which includes among its pillars the identification of needs that form the basis for the design and implementation of programmes of comprehensive PC [11].

There have been calls to prioritise PC research in LMIC, where a large proportion of the population could benefit significantly from it [12]. Researchers, research institutions and funding agencies have traditionally decided on the research question(s) to be asked [13]. However, influential health service bodies such as the World Health Organization (WHO) [14], and the United Kingdom’s National Institute for Health Research (NIHR) [15] are increasingly recommending the active involvement of non-research expert stakeholders in establishing research agendas, and research priority setting exercises based on their individual needs.

Research priority setting [16] involves any activity where stakeholders identify, prioritise, and reach agreement on subjects, issues, or questions they believe need to be addressed through research [17, 18]. This is particularly important at the beginning of the research process, prior to deciding what to research, and has been shown to increase the uptake and implementation of research evidence [19], and decrease “research waste” [20] by promoting the overall applicability and acceptability of research to policy and practice [17].

The implementation of integrated PC into cancer and non-cancer services has proven difficult despite a strong research evidence base [21, 22]. PC is also a sensitive, cultural issue, requiring a particular approach that considers diverse experiences, especially for those in LMIC such as Colombia. Acknowledging these cultural differences is essential for developing relevant health policies and clinical practice [12]. However, fundamental components of PC, such as the physical, psychological, spiritual and social needs of patients and informal carers; meanings tied to the illness; broader issues around end-of-life suffering, including decisions and preferences around place of death, are all under researched in LMIC [23, 24].

The Global Forum for Health Research [25] has condemned the huge gap in research output between LMIC and high-income countries (HIC), known at the 10/90 gap, representing the fact that under 10% of funds for health research are spent on health issues in LMIC, even though they have 90% of disease burden globally. A key strategy agreed by the Global Forum for Health Research was to encourage more research priority setting, an approach that we take in this paper to improve PC in Colombia.

## Methods

### Aim

The overall aim is to conduct a research PSP exercise to provide a service user and service provider informed roadmap for future PC research in Colombia.

### Objectives

1. Work with key stakeholders (service users and carers, allied health and social care providers (AHSCPs), and healthcare insurance providers) to identify PC research priorities in Colombia,
2. Work with key stakeholders to identify barriers and facilitators to PC research in Colombia.

### Study design

The PSP followed the James Lind Alliance (JLA) methodology which involves four stages (https://www.jla.nihr.ac.uk/jla-guidebook/downloads/JLA-Guidebook-Version-10-March-2021.pdf). This is the recommended framework in the Reporting guideline for priority setting of health research (REPRISE) [17].

### Setting and attendees

#### Stage 1: establishing the partnership

The first author, Tracey McConnell (TM) secured funding from Queen’s University Belfast (QUB) in 2022 to build on existing partnerships within Colombia and conduct this research priority setting exercise. Recognising the contextual differences between the UK and a middle-income country in South America, the key aim was to find out what the priority research questions and topics were for those requiring and providing PC in Colombia. TM established links with colleagues (co-authors, Joanne Reid (JR) and Gillian Prue (GP)) from the School of Nursing and Midwifery, QUB who had a history of working and publishing around improving palliative care with colleagues from Pontificia Universidad Javeriana (PUJ) and Universidad del Cauca (CU) in Colombia [22, 26]. This active PC working group was formed focusing on how to better integrate PC within the care continuum for cancer and non-cancer patients in Colombia.

### Data collection

#### Stage 2: identifying evidence uncertainties

Unanswered research questions for service users and carers, AHSCPs, and healthcare insurance providers were collated via Mentimeter during an online Microsoft Teams (MS Teams) PSP workshop held 25 January 2023. We held online seminars prior to this workshop to promote knowledge exchange and partnership building: The participants represented several regions of Colombia, representative of the country’s social and economic diversity. They included urban and rural universities, hospitals (PC providers and nurses), patients, carers and policy makers (PC observatory, Colombian League Against Cancer) to ensure the PC research priorities and recommendations for conducting the research are appropriate/suitable for the diverse Colombian contexts. The following broad questions were asked to help identify the evidence uncertainties: 1. What are the priority research questions for improving access to PC services in Colombia? 2. What are the challenges to conducting research in this area? 3. What are the possible solutions?

The second author, and international collaborator, Cindy Vanesa Mendieta (CVM) is bilingual and translated the questions from English to Spanish. When the exercise was completed, CVM translated the responses from Spanish to English for analysis. This translation was subsequently revised by additional bilingual international research partners (Esther de Vries (EdV) and Jose A Calvache (JAC)).

#### Stage 3: refining questions and uncertainties

This stage of the priority setting partnership involved an online survey using Microsoft Forms (see supplementary file 1) to refine initial questions and uncertainties. The survey had three key sections divided up in relation to research priorities, challenges and solutions to research in this area. Each section had a further list of questions developed from responses to the Stage 2 Mentimeter exercise. CVM again translated the questions from English to Spanish, and in the Mentimeter exercise they were presented in Spanish. Stakeholders were asked to rank the research questions/topics most important to them by using the arrow function or by clicking on the question/topic to move it up or down in order of what was most important, starting with the top priority down to the lowest priority.

#### Stage 4: prioritisation

Four final in-person PSP workshops took place in Bogota (capital city) and Popayan (small rural city) from the 13th to 16th February 2023. Two workshops were held in each urban and rural city: in the morning for service users and carers, and in the afternoon for AHSCPs as these times were most convenient for the two groups. The workshops adhered to the JLA methodology, which uses a Nominal Group Technique to encourage discussion, ranking and agreement by consensus.

The JLA method values creating an opportunity to prioritise the top questions via stakeholder discussion and knowledge exchange. The workshops were chaired and facilitated by CVM, EdV and JAC who are bilingual. CVM is a PhD student in Clinical Epidemiology with prior experience of facilitating research workshops. EdV is a Professor of Clinical Epidemiology with recent experience in qualitative studies in the field of palliative care in Colombia. JAC is a senior clinical academic in Epidemiology, Anaesthesiology and Pain Management/Palliative care. TM has prior experience of priority setting for health care research and oversaw the running of the workshops. A professional translator was also present to translate from Spanish to English for the QUB partners, and from English to Spanish for the participants. A QR code was generated for the MS form, and stakeholders could complete the ranking exercise either on their own devices or on a tablet provided by the research team. Stakeholders worked in small groups to rank the questions, discuss their decision making, and reach a consensus. This process was especially challenging for service users and carers as many of them had very low literacy levels and little understanding of what was meant by the term research. As such, their participation required individual support from the bilingual colleagues, the QUB team and translator. Given the challenges encountered during this first service user and carer workshop in Bogota, the service user and carer workshop in Popayan focused only on the first list of questions around the research priority topic area which was reframed as ‘what is most important to you in terms of improving your care?’ The second and third set of questions around challenges and solutions to research were removed as service users and carers really struggled with these topic areas in the first workshop. Participants were invited to provide verbal feedback on what they felt worked well, and on where improvements could be made at the end of each workshop. We chose not to use feedback forms due to the low literacy rates among the workshop participants.

### Data analysis

Data from the Mentimeter exercise were collated and used to inform the questions for the MS Form online and in-person prioritisation ranking exercise. The data from the MS Form prioritisation exercise were analysed using descriptive statistics.

## Results

A total of 33 stakeholders attended the online workshop and completed the Mentimeter exercise in MS Teams. The Mentimeter results from this workshop can be found in supplementary file 2, showing what stakeholders regarded as the most important unanswered questions in relation to palliative care in Colombia. The top PC research priority from this exercise was patient and family caregivers PC need.

A total of 48 attended the subsequent in person PSP exercise; 22 in Bogota and 26 in Popayan. The stakeholders were a diverse group of health professionals (physicians, medical students, nurses, dentists, physiotherapists, nutritionist, occupational and speech therapists), financial and administrative staff and patients with life-limiting illness and caregivers.

**Fig. 1:**
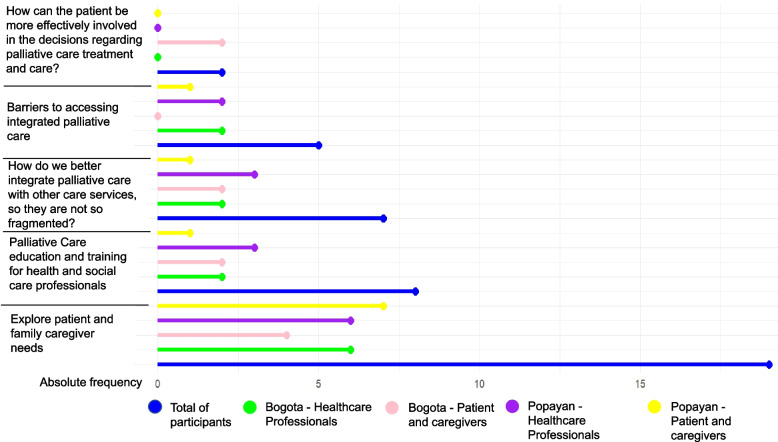
Priority research topics for improving access to palliative care services in Colombia

Figure 1 represents a high-level summary of the stakeholder’s priority research topics ranked in order of what is most important to them. Of the 48 stakeholders, three did not provide a response. All stakeholder groups (HCPs *n* = 10, patients and caregivers *n* = 9) in urban (Bogota) and rural (Popayan) settings considered the exploration of patient and family caregivers’ needs as the top research priority. Palliative care education and training for AHSCPs was the second priority topic for all stakeholder groups (HCPs *n* = 5, patients and caregivers *n* = 3), and again this was observed across urban and rural settings. Better integration of palliative care to address fragmentation between services came a close third in terms of research priorities, with slightly more support for this topic among stakeholders in Bogota (HCPs *n* = 2, patients and caregivers *n* = 3), compared to those in rural Popayan (HCPs *n* = 1, patients and caregivers *n* = 1). Fewer stakeholders overall rated understanding barriers to accessing palliative care as most important, with similar responses across both locations. Only two AHSCPs in Bogota ranked patient involvement in their palliative care treatment and care decisions as a top research priority. There were four remaining topic areas, including facilitators to integrated palliative care, barriers to communication, paediatric palliative care, and research in remote areas which were not ranked as priorities by any stakeholder groups.

**Fig. 2:**
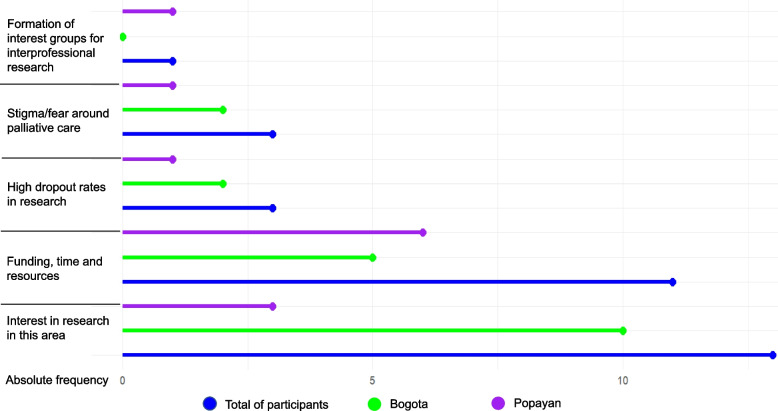
Challenges to conducting research in palliative care according to allied health and social care professionals

Figure 2 shows what AHSCP stakeholders rank as the main challenges to developing palliative care research in Colombia. There were no responses from patients and caregivers as they had little understanding of what was meant by the term research. Having a lack of interest in palliative care research was ranked as the main challenge by AHSCPs in urban and rural locations. This had a higher rate of responses from those in Bogota, but this could also be explained by the higher number of participating stakeholders (*n* = 22) compared to Popayan (*n* = 12). The next main challenge was about funding, time, and resources which were ranked similarly in urban and rural locations. Only three other challenges were rated as important by a low number of AHSCPs in both locations, and included high participant dropout rates and other difficulties in follow-up, stigma and fear around palliative care, and being able to form palliative care interprofessional special interest groups. Several issues identified in the first online priority-setting exercise were not deemed as priority challenges to address by in-person stakeholders. These included a lack of available data, conducting research with vulnerable populations, language/communication barriers, and having few qualified researchers.

**Fig. 3:**
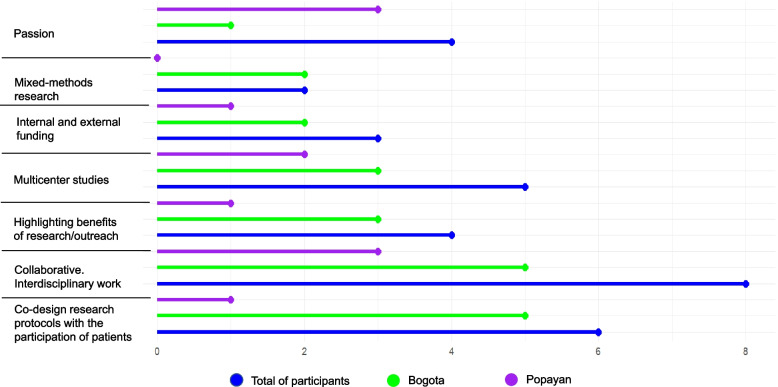
Possible solutions to conducting research in palliative care according to allied health and social care professionals

Figure 3 shows what AHSCP stakeholders rank as the main solutions for conducting palliative care research in Colombia. Collaborative and interdisciplinary work was highlighted as one of the main solutions in urban and rural locations. The second priority solution was to ensure research protocols were co-designed with patients, although this was viewed as a higher priority in urban Bogota (*n* = 5) compared to rural Popayan (*n* = 1). However, values such as passion were viewed as a higher priority solution in rural areas compared to urban areas. Having palliative care research studies across several centres, highlighting the benefits of palliative care research, having internal and external funding, and mixed method research were also ranked as priority solutions. Possible solutions that were not ranked as priorities included having a research mentor, methodological rigour, and research visibility.

### Feedback on the priority setting exercise

Feedback was positive overall, especially from service users who felt empowered by the opportunity to have their voice heard in relation to what is important to them in relation to their care. Service users did however report that they found it difficult to prioritize methods of research because of their lack of knowledge on research and the low literacy of the users and carers.

## Discussion

This research priority setting exercise provides a service user and service provider informed roadmap for future research in Colombia. The findings included the perspectives of an interdisciplinary group from rural and urban Colombia, which could be useful for PC organisations, research funders, and local governments to inform and develop PC research strategies and focus resources into what really matters to both service providers and service users with a potential for impact on policy making.

An overlap was noted between our research priorities and recommendations from a recent Delphi study by Paiva et al. in 2023 with 18 palliative care AHSCP experts focused on how to advance palliative care research in South America [27]. Top priorities from our work and their recommendations included the need for more collaborative PC research and practice, PC education and training and highlighting the benefits and importance of palliative care to funders, such as governmental bodies, to secure time and resources for research in this area.

It is unsurprising that PC education and training was one of the top priorities for research. A recent social mapping study involving a diverse stakeholder group such as AHSCP, patient, caregiver organisations across Colombia identified limited formal and specialised PC education and training as a key barrier to PC access [28]. Stakeholders in this study identified the need for PC education in both the undergraduate medical and nursing curriculum, and in postgraduate medical, nursing, social work and psychology education. In Colombia, the availability of PCs well-structured and specialized services are found in the main cities of the country while there are many regions without provision of PC. In addition, PC training programmes are not mandatory or available for health personnel or are provided mainly for medical and nursing programmes. Thus, PC training for AHSCP is even more limited [29]. Although intermediate (diploma courses) and advanced speciality level training courses in PC are offered by the PC society in Colombia for both adults and children, professionals must self-fund this training. This links closely with other top priorities from this priority setting partnership and the social mapping study [28], namely the need for more collaborative working, and highlighting the benefits and importance of PC. For example, PC education is crucial to address both of these key priorities as lack of understanding and knowledge of PC prevents joint working between AHSCPs, patient organisations and community care providers [29, 30]. Palliative education is also critical to address poor understanding and awareness of PC among the public, which in turn impacts on community support, and lack of awareness among the public about when PC [31] is needed. Previous studies have also supported the need for PC education among primary care providers, along with the importance of service providers, academic institutions, and policy makers highlighting the benefits of palliative care to the media and in policy areas in order to promote access to PC for those who need it [7, 30, 32].

The final top priority, in relation to better integration of palliative care, was also a key recommendation in the previous social mapping study. Again, this is not surprising given the fragmentation of PC in Colombia [30, 33]. This high fragmentation can be explained by the lack of specialized PC providers or lack of training on basic PC for primary care physicians or health care providers in rural areas, especially those in the most remote areas, deemed to have high risk safety issues for health personnel [32, 33]. This further highlights the need to lobby for the importance of PC to health insurance providers to improve PC coverage in Colombia, and to government bodies so they see the need to improve the safety of healthcare providers in remote areas [28]. Improving early detection and initiation of PC, even in rural areas by accessing basic PC, may reduce costs while positively increasing the quality of life and decision making in patients with life-limiting diseases [34–37]. Through timely symptom management, psychosocial support, and Advance Care Planning, individuals may experience fewer hospitalizations, emergency room visits, and unnecessary medical interventions, thereby alleviating the financial burden associated with prolonged and aggressive treatments. Moreover, by integrating palliative care into the continuum of care from the outset of illness and starting PC in rural areas by primary care physicians or trained health care providers, patients and their families are empowered to make informed decisions aligned with their needs, values, preferences, and goals.

In terms of enabling PC research, Paiva et al.’s Delphi recommendations were similar to those of our stakeholders, who saw establishment of multicenter studies as a priority, to support greater research collaboration not only between institutes, but also across countries, and disciplines, which may also go some way to addressing fragmentation of services. Previous research has provided further support for this priority, demonstrating that multicenter studies with international collaborators tend to have higher citations, greater participation, more generalisable results, and stronger research designs compared to those without international collaboration [38].

Our research priority partnership is to our knowledge, the first to include the top priorities for AHSCPs, patients and carers in South America, specifically Colombia which is among the lowest ranking countries for quality of death and dying [31, 39]. We identified priorities not included in the recommendations from the previous Delphi study. For example, patient and family caregiver needs were rated as the top priority overall and highlights the importance of including service users as well as service providers in this type of exercise to ensure priorities address the needs of those services are designed for [14, 15]. Similarly, one of the top solutions for developing palliative care research in Colombia was co-production of research with patients from the early protocol development stage, which has been supported in previous research as a key strategy for producing research to meet patient need in real-world practice [40, 41].

Our research team are now actively planning a programme of research to address patient needs in Colombia through feasibility testing of the recently translated (into Spanish) and cross-culturally validated SPARC holistic patient needs assessment tool for use in Colombia—SPARC-Sp [42, 43].

### Strengths and limitations

Limitations of this study include a limited geographical and cultural coverage of stakeholders: only stakeholders from the regions close to Popayan and Bogota could participate in the on-site workshops. However, in the first online rounds there were also participants from other parts of the country. Unfortunately, it was not feasible to include the research methods prioritisation exercise with patients and caregivers, for a lack of understanding amongst most of them on what research is – and therefore hampering an evaluation of gaps and recommendations for improvements. This however, is also a finding in itself: in Colombia, patient and public involvement in research is in its infancy [26] and many people in Colombia have low literacy rates. This, in combination with the little research being done in the area of palliative care, makes it impossible for patients and caregivers to understand and therefore prioritise. This lack of understanding about research, and low literacy should be taken into consideration to support involvement of service users in PC research in Colombia [26] Strengths of this study include the diverse backgrounds of the participating stakeholders, moreover from both large urban and more rural and remote settings. The fact that patients and caregivers were given a voice in this study is an important novelty – their voices are largely overlooked in Colombia and Latin America in general.

### Implications

The findings of this PSP exercise could be disseminated among PC associations worldwide, with similar characteristics to the Colombian context, to inform international multicentre studies, and among governmental, nongovernmental organisations and funding bodies that promote research in Colombia. A focus on patient and family caregiver palliative care needs in Colombia should be prioritised by researchers, funding bodies and current policies.

## Conclusions

Research priorities to improve palliative care in Colombia were identified using a priority setting partnership approach. Top priorities included patient and caregiver needs, service provider education and training, integration of palliative care and collaboration across disciplines and settings, highlighting benefits of palliative research to help secure adequate resources, and multicentre, mixed method research involving patients throughout.

### Supplementary Information

Supplementary Material 1.

Supplementary Material 2.

## Data Availability

Data are provided within the manuscript or supplementary information files.

## Abbreviations

PC: Palliative Care
AHSCPs: Allied Health and Social Care Professionals
