# Supplementary material for: Developing research priorities for palliative care in Colombia: a priority setting partnership approach
Source: BMC Palliat Care. 2024 Aug 1;23:194. doi: 10.1186/s12904-024-01534-z (PMC11295305; doi:10.1186/s12904-024-01534-z)
Supplement: Supplementary file 1 — Supplementary Material 1. [file 12904_2024_1534_MOESM1_ESM.docx]

**Improving access to palliative care in Colombia**

1. Which of the following research questions/topics are most important to you? (Use the arrows or click on the option and move it up or down in order of what is most important to you, starting with the most important at the top down to least important)

| Explore patient and family caregiver needs |
| --- |
| How do we better integrate palliative care with other care services so they are not so fragmented? |
| Barriers to accessing integrated palliative care |
| Facilitators for Integrated Palliative and Curative Care |
| Measuring the impact of integrating palliative care with the other care services |
| Barriers to communication |
| Palliative Care education and training for health and social care professionals |
| Paediatric palliative care |
| Research in remote areas |
| How can the patient be more effectively involved in the decisions regarding palliative care treatment and care? |

1. What are the challenges to research (gaining new knowledge) in this area? (Use the arrows or click on the option and move it up or down in order of what you think is most important – starting with the most important at the top down to the least important)

| Funding, time and resources |
| --- |
| Interest in research in this area |
| Qualified researchers |
| Stigma/fear around palliative care |
| Language/communication barriers |
| High dropout rates in research |
| Vulnerable population |
| Amount of information |
| Formation of interest groups for interprofessional research |
| Lack of data availability |

1. What could facilitate research (gaining new knowledge) in this area? (Use the arrows or click on the option and move it up or down in order of what you think is most important – starting with the most important at the top down to the least important)

| Collaborative. Interdisciplinary Work |
| --- |
| Multicenter studies |
| Mixed-methods research |
| Co-design research protocols with the participation of patients |
| Internal and external funding |
| Passion |
| Research visibility |
| Highlighting benefits of research/outreach |
| Methodological rigour |
| Mentoring |
