# Supplementary material for: Developing research priorities for palliative care in Colombia: a priority setting partnership approach
Source: BMC Palliat Care. 2024 Aug 1;23:194. doi: 10.1186/s12904-024-01534-z (PMC11295305; doi:10.1186/s12904-024-01534-z)
Supplement: Supplementary file 2 — Supplementary Material 2. [file 12904_2024_1534_MOESM2_ESM.docx]

1. **¿Cuáles son las preguntas de investigación prioritarias para integrar los cuidados paliativos con la oncología en Colombia?**

- **What are the priority research questions for integrating palliative care with oncology in Colombia?**


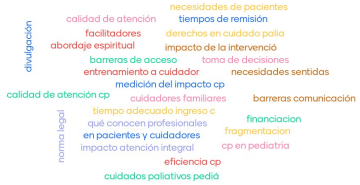


| **Original** | **Translation** |
| --- | --- |
| Necesidades de pacientes - Necesidades sentidas | Patient needs - Felt needs |
| Derechos en cuidado paliativo | Rights in palliative care |
| Tiempo adecuado ingreso oportuno - Tiempos de remisión | Adequate time for timely admission - Referral times |
| Fragmentación | Fragmentation |
| Impacto de la intervención - Impacto atención integral - Medición del impacto en cuidados paliativos | Impact of intervention - Impact of integrated care - Measuring palliative care impact |
| Barreras de comunicación - Barreras de acceso | Barriers to communication - Barriers to access |
| Facilitadores | Facilitators |
| Abordaje espiritual | Spiritual approach |
| Entrenamiento a cuidador | Caregiver training |
| Eficiencia cuidado paliativo | Efficiency of palliative care |
| Calidad de atención - Calidad de atención en cuidados paliativos | Quality of care - Quality of care in palliative care |
| Toma de decisiones | Decision-making |
| Cuidadores familiares | Family caregivers |
| Cuidados paliativos en pediatría - Cuidados paliativos pediátricos | Palliative care in paediatrics - Paediatric palliative care |
| Divulgación | Dissemination |
| En pacientes y cuidadores | In patients and caregivers |
| Qué conocen profesionales | What did professionals know? |
| Normal legal | Legal standard |
| Financiación | Funding |

1. **¿Cuáles son los desafíos para realizar una investigación?**

- **What are the challenges in conducting research?**


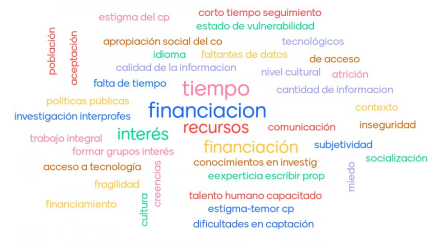


| **Original** | **Translation** |
| --- | --- |
| Financiación – Financiamiento | Financing - Financing |
| Tiempo | Time |
| Recursos | Resources |
| Interés | Interest |
| Faltantes de datos | Data gaps |
| Políticas públicas | Public policy |
| Fragilidad | Fragility |
| Contexto | Context |
| Apropiación social del conocimiento | Social appropriation of knowledge |
| Acceso – Acceso a tecnología | Access - Access to technology |
| Conocimientos en investigación – Talento humano capacitado | Knowledge in research - Trained human talent |
| Corto tiempo seguimiento | Short follow-up time |
| Población | Population |
| Aceptación | Acceptance |
| Comunicación | Communication |
| Estigma del cuidados paliativos - Estigma – temor cuidados paliativos – miedo - inseguridad | Palliative care stigma - Stigma - Fear palliative care - Fear - Insecurity |
| Atrición | Atrition |
| Trabajo integral – formar grupos de interés – Investigación interprofesionales | Integral work - forming interest groups - Interprofessional research |
| Creencias | Beliefs |
| Tecnológicas | Technological |
| Calidad de la información | Quality of information |
| Nivel cultural | Cultural level |
| Cantidad de información | Quantity of information |
| Subjetividad | Subjectivity |
| Dificultades en capacitación | Difficulties in training |
| Estado de vulnerabilidad | Vulnerability status |
| Idioma | Language |
| Interés | Interest |
| Socialización | Socialisation |
| Experticia escribir propuestas | Expertise in writing proposals |
| Cultura | Culture |

1. **¿Cuáles son las posibles soluciones?**

- **What are the possible solutions?**


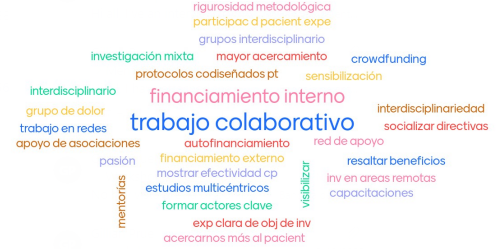


| **Original** | **Translation** |
| --- | --- |
| Trabajo colaborativo | Collaborative work |
| Financiamiento interno | Internal funding |
| Participación del paciente experto | Expert patient involvement |
| Sensibilización | Awareness raising |
| Grupo de dolor | Pain group |
| Financiamiento externo | External funding |
| Protocolos codiseñados con pacientes | Protocols co-designed with patients |
| Interdisciplinariedad | Interdisciplinarity |
| Apoyo de asociaciones | Support from associations |
| Mentorías | Mentoring |
| Mayor acercamiento | Greater outreach |
| Socializar directivas | Socialising guidelines |
| Autofinanciamiento | Self-financing |
| Exposición clara de objetivo de investigación | Clear statement of research objective |
| Rigurosidad metodológica | Methodological rigour |
| Red de apoyo | Support network |
| Investigación en áreas remotas | Research in remote areas |
| Acercarnos más al paciente | Getting closer to the patient |
| Crowfunding | Crowfunding |
| Trabajo en redes | Networking |
| Pasión | Passion |
| Resaltar beneficios | Highlighting benefits |
| Mostrar efectividad de cuidados paliativos | Showing effectiveness of palliative care |
| Estudios multicéntricos | Multicentre studies |
| Grupos interdisciplinario | Interdisciplinary groups |
| Capacitaciones | Training |
| Investigación mixta | Mixed research |
| Interdisciplinario | Interdisciplinary |
| Visibilizar | Visibilise |
| Formar actores clave | Training key actors |
